# Dorsal recruitment with flow-controlled expiration (FLEX): an experimental study in mechanically ventilated lung-healthy and lung-injured pigs

**DOI:** 10.1186/s13054-018-2168-9

**Published:** 2018-09-29

**Authors:** Silke Borgmann, Johannes Schmidt, Ulrich Goebel, Joerg Haberstroh, Josef Guttmann, Stefan Schumann

**Affiliations:** 1 Department of Anesthesiology and Critical Care, Medical Center, University of Freiburg, Hugstetter Str. 55, 79106 Freiburg, Germany; 2 Experimental Surgery, Center for Experimental Models and Transgenic Service, Medical Center, University of Freiburg, Breisacher Str. 66, 79106 Freiburg, Germany; 3 Faculty of Medicine, University of Freiburg, Freiburg, Germany

**Keywords:** Acute respiratory distress syndrome, Electrical impedance tomography, Oleic acid, Positive pressure ventilation, Expiration control

## Abstract

**Background:**

Concepts for optimizing mechanical ventilation focus mainly on modifying the inspiratory phase. We propose flow-controlled expiration (FLEX) as an additional means for lung protective ventilation and hypothesize that it is capable of recruiting dependent areas of the lungs. This study investigates potential recruiting effects of FLEX using models of mechanically ventilated pigs before and after induction of lung injury with oleic acid.

**Methods:**

Seven pigs in the supine position were ventilated with tidal volume 8 ml·kg^− 1^ and positive end-expiratory pressure (PEEP) set to maintain partial pressure of oxygen in arterial blood (paO_2_) at ≥ 60 mmHg and monitored with electrical impedance tomography (EIT). Two ventilation sequences were recorded - one before and one after induction of lung injury. Each sequence comprised 2 min of conventional volume-controlled ventilation (VCV), 2 min of VCV with FLEX and 1 min again of conventional VCV. Analysis of the EIT recordings comprised global and ventral and dorsal baseline levels of impedance curves, end-expiratory no-flow periods, tidal variation in ventral and dorsal areas, and regional ventilation delay index.

**Results:**

With FLEX, the duration of the end-expiratory zero flow intervals was significantly shortened (VCV 1.4 ± 0.3 s; FLEX 0.7 ± 0.1 s, *p* < 0.001), functional residual capacity was significantly elevated in both conditions of the lungs (global: healthy, increase of 87 ± 12 ml, *p* < 0.001; injured, increase of 115 ± 44 ml, *p* < 0.001; ventral: healthy, increase of 64 ± 11 ml, *p* < 0.001; injured, increase of 83 ± 22 ml, *p* < 0.001; dorsal: healthy, increase of 23 ± 5 ml, *p* < 0.001; injured, increase of 32 ± 26 ml, *p* = 0.02), and ventilation was shifted from ventral to dorsal areas (dorsal increase: healthy, 1 ± 0.5%, *p* < 0.01; dorsal increase: injured, 6 ± 2%, *p* < 0.01), compared to conventional VCV. Recruiting effects of FLEX persisted during conventional VCV following FLEX ventilation mostly in the injured but also in the healthy lungs.

**Conclusions:**

FLEX shifts regional ventilation towards dependent lung areas in healthy and in injured pig lungs. The recruiting capabilities of FLEX may be mainly responsible for lung-protective effects observed in an earlier study.

## Background

A special focus of current research in intensive care medicine and anesthesiology is on strategies for lung-protective mechanical ventilation. Current concepts include variation of end-inspiratory volume or peak or driving pressure [1–3]. However, except for positive end-expiratory pressure (PEEP), which certainly effects inspiration as well, little attention has been drawn so far on modifying the expiratory phase of the ventilation cycle. Consequently, in routine mechanical ventilation the expiration is guided solely by the passive recoil forces of the patients’ respiratory system [4]. Thus, for a patient suffering from acute respiratory distress syndrome (ARDS), the injured respiratory system determines the processing of the expiration phase, leading to high expiratory peak flow rates and rapid lung deflation as a consequence of the short time-constant of passive expiration. In addition to the high flow rates, airway pressure drops quickly during passive expiration and the lungs stay at PEEP level until the next inspiration cycle starts. By contrast, flow-controlled expiration (FLEX) decelerates the initial expiratory flow in favor of a moderate flow persisting throughout nearly the whole expiration phase [5]. It has been found that FLEX results in lower PEEP requirement, improved dynamic lung compliance and could better attenuate experimental ARDS compared to conventional volume-controlled ventilation (VCV) in a porcine lung injury model [5]. In addition, decelerating the expiratory flow improves the homogeneous distribution of ventilation in lung-healthy patients [6] and pigs [7]. In the light of these findings we hypothesized that FLEX affects the distribution of regional ventilation and provides additional recruiting effects. Therefore, we assessed the regional ventilation using electrical impedance tomography (EIT) during mechanical ventilation in the volume-controlled mode with and without FLEX in a pig model before and after induction of lung injury.

## Methods

The study was approved by the Animal Welfare Committee of the University of Freiburg and carried out in compliance with the German animal protection law and the European Union Directive on the protection of animals used for scientific purposes (2010/63).

### Preparation

In total, seven healthy German landrace hybrid pigs (bodyweight 62.5 ± 5.0 kg (mean ± SD), either sex) were included in the study. Each pig was fasted for 8 h and premedicated with 0.5 mg·kg^− 1^ midazolam (Dormicum, Roche, Grenzach- Wyhlen, Germany) and 20 mg·kg^− 1^ ketamine hydrochloride (Ketamin 10%, Intervet, Unterschleißheim, Germany). Anesthesia was induced by intravenous (i.v.) administration of 2 mg·kg^− 1^ propofol (Propofol 1%, Fresenius Kabi, Bad Homburg, Germany) and sustained with infusions of 1–2 mg·kg^− 1^·h^− 1^ midazolam, 4–6 mg·kg^− 1^·h^− 1^ ketamine hydrochloride and 10 μg·kg^− 1^·h^− 1^ fentanyl citrate (Fentanyl Janssen, Janssen-Cilag, Neuss, Germany). Vecuronium 0.5 mg·kg^− 1^·h^− 1^ (Vecuronium-Inresa, Inresa, Freiburg, Germany) was administered as a neuro-muscular blocking agent. Volume-controlled ventilation (Evita 4, Dräger Medical, Lübeck, Germany) was started after intubation with a respiratory frequency of 15 breaths per minute, tidal volume 8 ml·kg^− 1^ and PEEP 8 cm H_2_O. The I:E ratio was set to 1:1.5 and inspired oxygen fraction (FIO_2_) was kept at 0.21. All pigs were kept in the supine position throughout the procedure. For electrical impedance tomography (EIT) recordings (EIT Evaluation KIT II, Dräger Medical, Lübeck, Germany), each pig was equipped with an electrode belt placed transversally around the mid thorax.

### Protocol

Before lung injury was induced, an EIT sequence of 5 min (sampling rate 20 frames per second) was recorded. The sequence comprised 2 min of conventional VCV (VCV1 period), 2 min of VCV with FLEX (FLEX period) and again 1 min of conventional VCV (VCV2 period). After lung injury was established, another identical EIT sequence was recorded.

### Lung injury

Lung injury was obtained by repeated i.v. bolus administration of oleic acid emulsion with glucose solution (ratio 1:1, Oleic acid PhEur, 75,096, Sigma Aldrich, Munich, Germany and Glucose 5%, B. Braun Melsungen AG, Melsungen, Germany) until the ratio of partial pressure of oxygen in arterial blood (PaO_2_)/fraction of inspired oxygen (FiO_2_) was below 200 mmHg at an FIO_2_ of 1.0. Following the Acute Respiratory Distress Syndrome Clinical Network (ARDSnet) recommendations, PEEP and FiO_2_ were tuned such as to maintain a PaO_2_ > 60 mmHg: in such a case, first FiO_2_ was reduced to 0.8 and subsequently PEEP was reduced stepwise by 2 cm H_2_O in order to keep absorption atelectasis and peak pressure at a minimum.

### Flow-controlled expiration

**Fig. 1:**
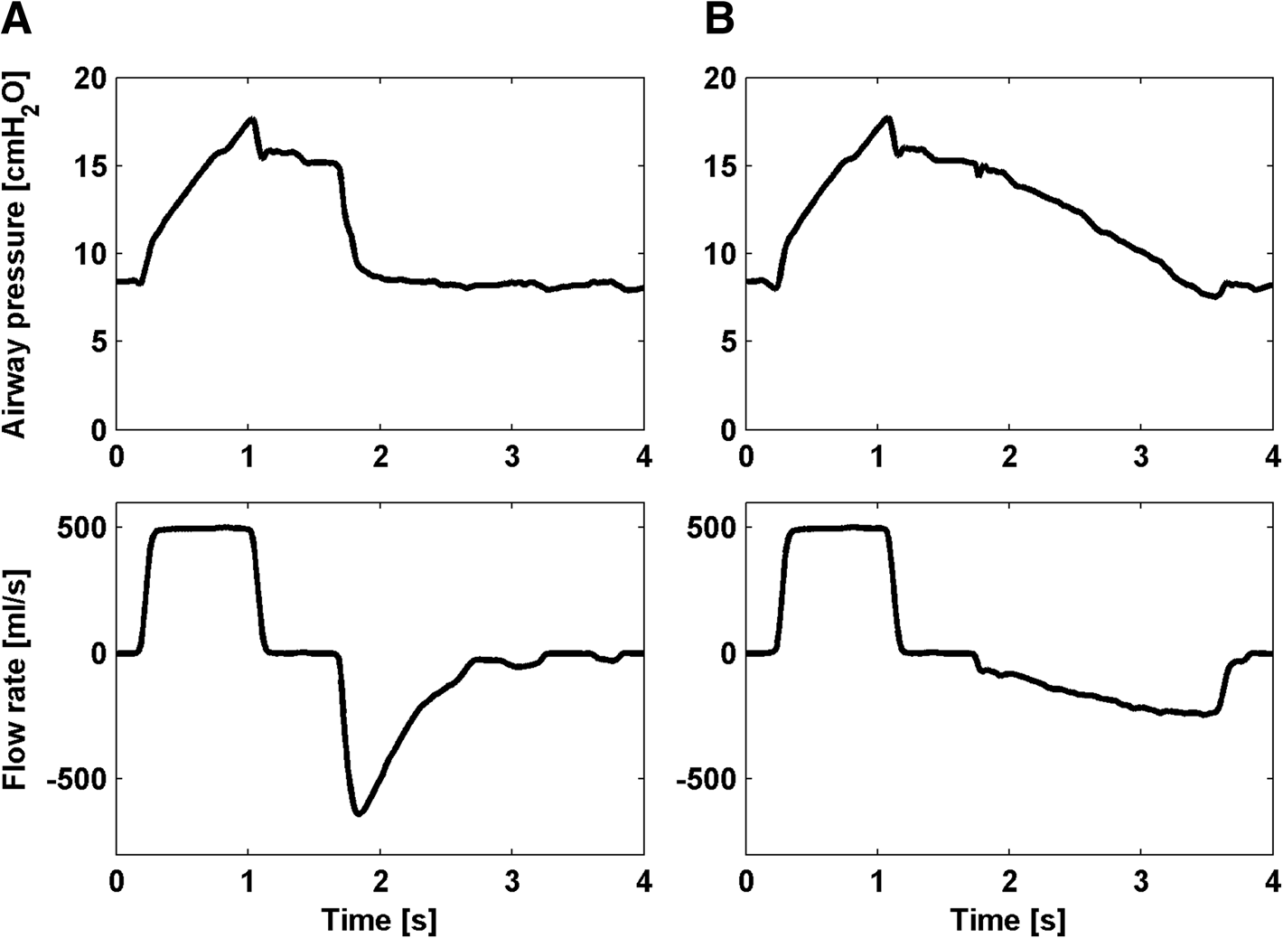
Flow and pressure time curves of representative breaths with volume-controlled ventilation (**a**) and volume-controlled ventilation with flow-controlled expiration (**b**)

FLEX was based on a controlled partial occlusion of the ventilator’s expiratory outlet. Therefore, for variable resistivity a cone was positioned inside the outlet aperture via a computer controlled linear motor (PS01-23Sx80, LinMot, Spreitenbach, Switzerland) [8]. During inspiration the cone was positioned to nearly occlude the aperture while during expiration the cone was continuously retracted from the aperture leading to a continuously decreasing resistance. Thus, the expiratory flow rate could be adjusted to follow a linear profile (Fig. 1). Flow and airway pressure data provided by the ventilator were recorded during all EIT recordings (sampling rate 125 Hz). The applied tidal volume during the EIT sequences was calculated in an offline analysis via integration of the flow data. Tracheal pressure was calculated from measured airway pressure (Paw) and flow rate considering the resistance coefficients of the endotracheal tube [9]. Dynamic compliance was calculated using multiple linear regression analysis [10]. As auto-PEEP may have potentially influenced our measurements, an auto-PEEP estimation was done using the method of Eberhard and colleagues [11]. In short, a least square fit of the equation of the classical resistance-compliance model of Brody [12] and Otis [13] was done, using data of the lowest 20% volume range of the pressure/volume (PV) loop. The difference between the dynamic pressure base (P_0_) and PEEP was assumed to give an estimate for auto-PEEP, if positive.

### Electrical impedance tomography

EIT recordings were evaluated using software developed in Matlab (MATLAB R2014a, The Mathworks Inc., Natick, MA, USA). As a first step, the relevant lung area was determined for each animal by applying the lung area estimation method to the recordings made prior to lung injury [14, 15]. In brief, for each pixel of an EIT frame the standard deviation over the whole recording was calculated as a measure of tidal ventilation change within this pixel and the pixel with maximal tidal variation was determined. Pixels with a standard deviation of more than 20% of this maximum were considered to contribute to the lung area. These pixels were then mirrored left to right, to ensure full lung coverage including atelectatic areas that were possibly already present in the analysis.

Subsequently, two sets of impedance curves and images were extracted from the EIT recordings: global and regional impedance curves and tidal variation images were calculated to illustrate the tidal variations over time. The global and regional impedance curves represent the sum of impedances of all pixels per frame or respectively per region of interest over time. The relation between tidal impedance peaks and tidal volume was used to scale the absolute impedance values of the EIT data (which depend on the calibration of the device and are arbitrary) to milliliters, rendering the data more intuitive and convenient [16].

The tidal variation images were generated by breath-by-breath subtraction of the frames representing beginning of inspiration from those representing end of inspiration. These differential images hence visualize the distribution of the ventilation during one breath in the cross-section of the lung monitored with EIT. The tidal images of all breaths of the investigated ventilation periods (VCV1, FLEX and VCV2) were then averaged resulting in mean tidal variation images for VCV1, FLEX and VCV2 for both the lungs in healthy and injured status, respectively.

After these preparative steps, the analyses were threefold. First, the global and regional impedance curves were analyzed with regard to differences between sequences of conventional VCV and VCV with FLEX. The baselines of tidal impedance curves, which correspond to a fraction of the functional residual capacity (FRC), were determined for the whole lung and separately for the ventral and dorsal regions of the lung. Furthermore, the duration of the end-expiratory intervals where no flow is present (zero-flow period) and the loss of impedance (δZ) during the zero-flow period were determined. Second, for illustrating the relative ventilation distribution in the two regions of interest the fractions of tidal impedance variation [17] in ventral (TV_v_) and dorsal (TV_d_) parts of the lung were determined by summing the impedance values of the mean tidal variation images in the respective region of interest (DI_v_, ventral and DI_d_, dorsal) and referencing them to the sum of impedances of the whole lung area DI_x,y_ (eq. 1):

1 $$ {\mathrm{TV}}_{\mathrm{v}}=\sum \frac{{\mathrm{DI}}_{\mathrm{v}}}{{\mathrm{DI}}_{\mathrm{x},\mathrm{y}}}\kern0.28em \mathrm{and}\kern0.28em {\mathrm{TV}}_{\mathrm{d}}=\sum \frac{{\mathrm{DI}}_{\mathrm{d}}}{{\mathrm{DI}}_{\mathrm{x},\mathrm{y}}} $$

Third, the regional ventilation delay (RVD) [18] was calculated to illustrate the time lag of inspiration for each pixel. The calculation of the RVD was adapted to allow a breath by breath determination of the ventilation delay. For this purpose, the lag of starting time of inspiration of each pixel was determined with respect to the pixel in which inspiration started earliest. Thus, the RVD is a visualization of ventilation delay within the lung and illustrates the ventilation dynamic within an average breath.

### Statistical evaluation

All data are presented as mean value ± standard deviation unless indicated otherwise. Statistical analysis was done using Matlab (R2014, The MathWorks Inc., Natick, MA, USA). The Lilliefors test was used to check for normal distribution on all data sets that were analyzed. Linear mixed effects analysis was performed to check for differences between the relevant cases using R software (R Core Team, 2017 and lme4, Bates, Maechler, Bolker and Walker, 2015). A level of *p* < 0.05 was considered statistically significant.

## Results

### Respiratory data

Expiratory peak flow was lower and mean tracheal pressure higher with FLEX in the healthy (both *p* < 0.001) and in the injured lungs (both *p* < 0.001) compared to VCV (Table 1). Tidal volume was similar in all cases and ventilation modes. Mean tracheal pressure did not differ between VCV2 and VCV1. The difference between P_0_ and PEEP was − 0.5 ± 0.4 cmH_2_O for VCV, and − 0.2 ± 0.5 cmH_2_O for FLEX with healthy lungs and − 1.1 ± 1.1 cmH_2_O for VCV and − 0.9 ± 1.6 cmH_2_O for FLEX with injured lungs.

**Table 1:** Summary of the respiratory data during VCV1, FLEX and VCV2

	VCV 1	FLEX	VCV 2
Healthy lung			
Compliance (ml·cm H_2_O^− 1^)	53 ± 6	51 ± 5	52 ± 5
Flow_Peak exp_ (ml s^− 1^)	− 669 ± 70	− 261 ± 12^*^	− 679 ± 64^*^
PEEP (cm H_2_O)	8	8	8
P_plat_ (cm H_2_O)	16 ± 1	17 ± 1	16 ± 1
P_mean_ (cm H_2_O)	12.3 ± 0.5	13.7 ± 0.5^*^	12.4 ± 0.4
V_T_ (ml)	419 ± 0.1	418 ± 0.2	418 ± 0.1
Zero flow time (s)	1.3 ± 0.1	0.5 ± 0.1^*^	1.3 ± 0.1
Injured lung			
Compliance (ml·cm H_2_O^−1^)	17 ± 3	16 ± 3	17 ± 3
Flow_Peak exp_ (ml s^− 1^)	− 970 ± 162	− 303 ± 11^*^	− 968 ± 161
PEEP (cm H_2_O)	12 ± 2	12 ± 2	12 ± 2
P_plat_ (cm H_2_O)	37 ± 3	39 ± 2	36 ± 3
P_mean_ (cm H_2_O)	24.6 ± 1.6	28 ± 3^ǂ^	24.2 ± 1.7
V_T_ (ml)	418 ± 0.2	418 ± 0.3	418 ± 0.2
Zero flow time (s)	1.4 ± 0.3	0.7 ± 0.1^*^	1.4 ± 0.3

Data are given as mean SD

*FLEX* flow-controlled expiration, *Flow*_*Peak exp*_ maximum expiratory flow, *PEEP* positive end-expiratory pressure, *P*_*mean*_ mean tracheal pressure, *P*_*plat*_ plateau pressure, *VCV* volume-controlled ventilation *V*_*T*_ tidal volume

*Statistically significant

### Global and regional impedance curves

**Fig. 2:**
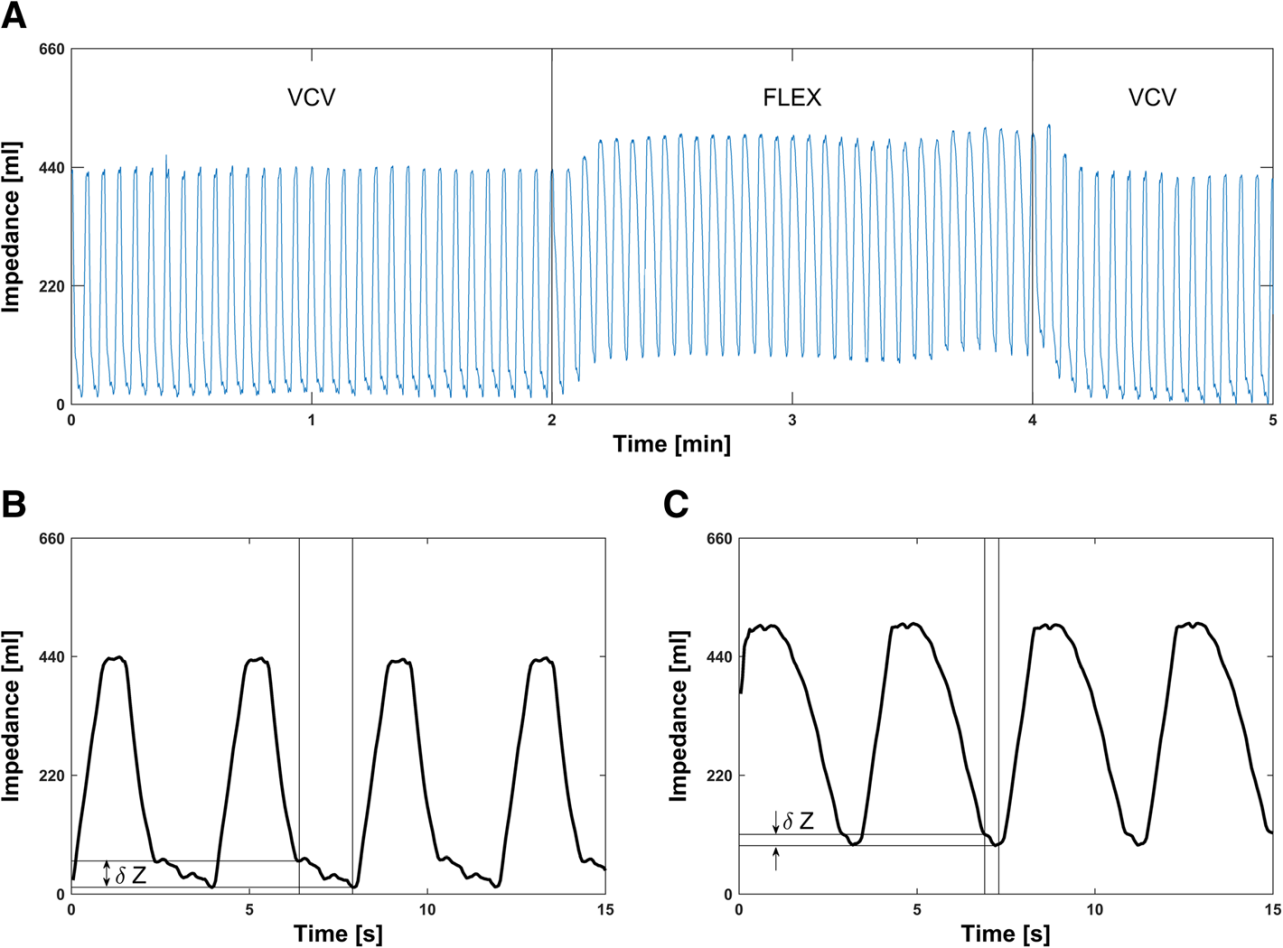
**a** Global impedance curve of pig number 7 before established lung injury. To help visualization, the different ventilation modes are separated by vertical lines. The elevation of the baseline of the global impedance curve during flow-controlled expiration (FLEX) was significant (*p* < 0.001) for all pigs regardless of whether the lung was healthy or injured. The local impedance curves representing the ventral and dorsal areas of the lung showed a similar significant elevation under FLEX ventilation. **b**, **c** Sequence of several breaths extracted from the global impedance curve of the electrical impedance tomography recordings of pig number 1. In **b**, breaths using volume-controlled ventilation (VCV1) are depicted. In **c**, breaths during usage of FLEX are shown. The time of no flow was significantly reduced under FLEX (*p* < 0.001) and the associated impedance decrease δZ was significantly less (*p* = 0.04) than under VCV only

In both conditions of the lungs, healthy and injured, the baselines of the global impedance curves (Fig. 2a) increased whenever FLEX was switched on (healthy, increase of 87 ± 12 ml, *p* < 0.001; injured, increase of 115 ± 44 ml, *p* < 0.001). The global baseline between VCV1 and VCV2 was not shifted significantly in the healthy lungs (difference − 4 ± 8 ml, *p* = 0.17) whereas in the injured lungs, the baseline was elevated by 39 ± 27 ml during VCV2 compared to VCV1 (*p* < 0.01). With FLEX the baselines of the regional impedance curves in the healthy and in the injured lungs were both elevated in the ventral (healthy, increase of 64 ± 11 ml, *p* < 0.001; injured, increase of 83 ± 22 ml, *p* < 0.001) and in the dorsal (healthy, increase of 23 ± 5 ml, *p* < 0.001; injured, increase of 32 ± 26 ml, *p* = 0.02) regions. In the healthy lungs, the regional baselines differed only in the dorsal region (difference, ventral 0 ± 8 ml, *p* = 0.97; difference, dorsal − 5 ± 5 ml, *p* = 0.04) between VCV1 and VCV2, whereas in the injured lungs the baselines during VCV2 were elevated by 20 ± 13 ml (*p* < 0.001) in the ventral and by 19 ± 17 ml (*p* = 0.02) in the dorsal region compared to VCV1.

In the injured animals, the zero-flow period at the end of expiration was significantly reduced during FLEX ventilation in all cases (VCV 1.4 ± 0.3 s and FLEX 0.7 ± 0.1 s, *p* < 0.001). The associated loss of impedance (δZ, Fig. 2b,c) was lower during FLEX ventilation than during VCV (− 49 ± 24 ml (VCV); − 33 ± 18 ml (FLEX), *p* = 0.04).

### Ventral and dorsal tidal variation

**Fig. 3:**
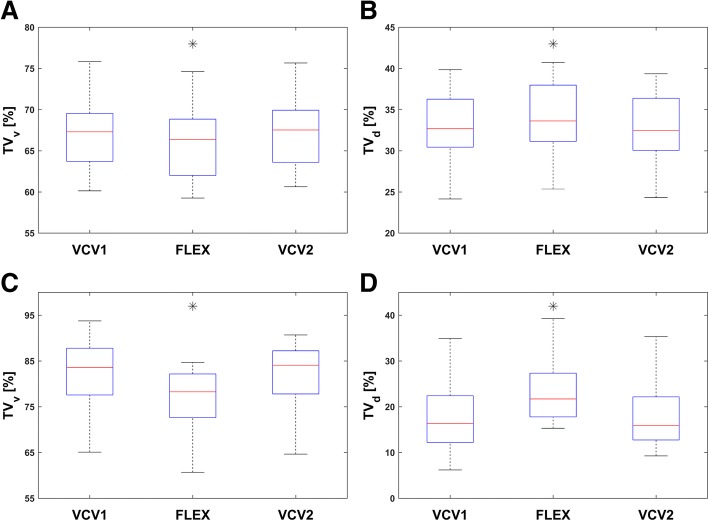
Fractional tidal impedance variation for the healthy (**a**) and (**b**) and the injured lung (**c**) and (**d**). In general, the main part of the ventilation is located in the ventral area (66.7 ± 4.9%). However, during usage of FLEX a significant redistribution of ventilation from ventral to dorsal areas can be seen in the healthy (*p* = 0.002) as well as the injured lung (*p* < 0.001)

The fractional tidal impedance variation showed a larger fraction of ventilation in the ventral region of the lungs (66.7 ± 4.9%) during VCV1 and VCV2 in both lung conditions, healthy and injured (Fig. 3). However, when switching to FLEX, TV_v_ decreased and TV_d_ increased in the healthy (*p* = 0.002) and in the injured lungs (*p* < 0.001). No significant differences in TV_v_ and TV_d_ were found between VCV1 and VCV2 (healthy, *p* = 0.15; injured, *p* = 0.23).

### Regional ventilation delay

**Fig. 4:**
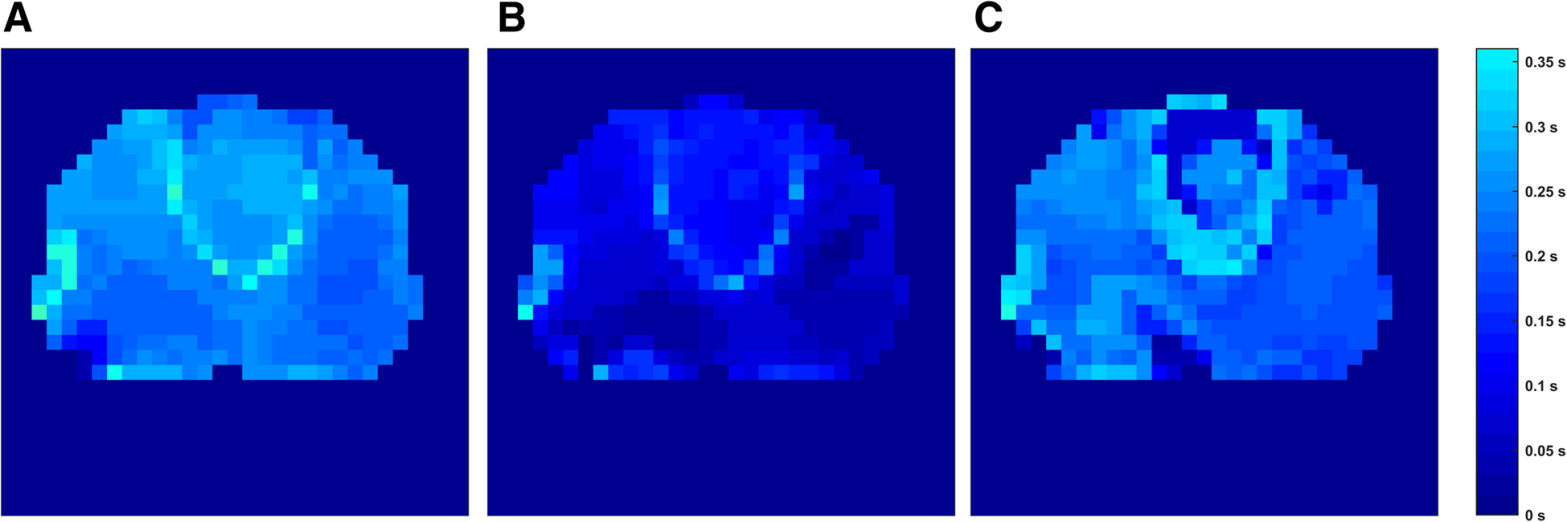
Regional ventilation delay for the healthy lung under volume-controlled ventilation (VCV) (**a**), flow-controlled expiration (FLEX) ventilation (**b**) and second VCV period (**c**). Colder (darker blue) colors represent lung areas that participate early in the ventilation, warmer (lighter blue) colors are areas with a delay in inspiration starting time. A larger fraction of lung area participates earlier in ventilation when using FLEX ventilation. This effect is nearly mitigated after switching off FLEX ventilation

**Fig. 5:**
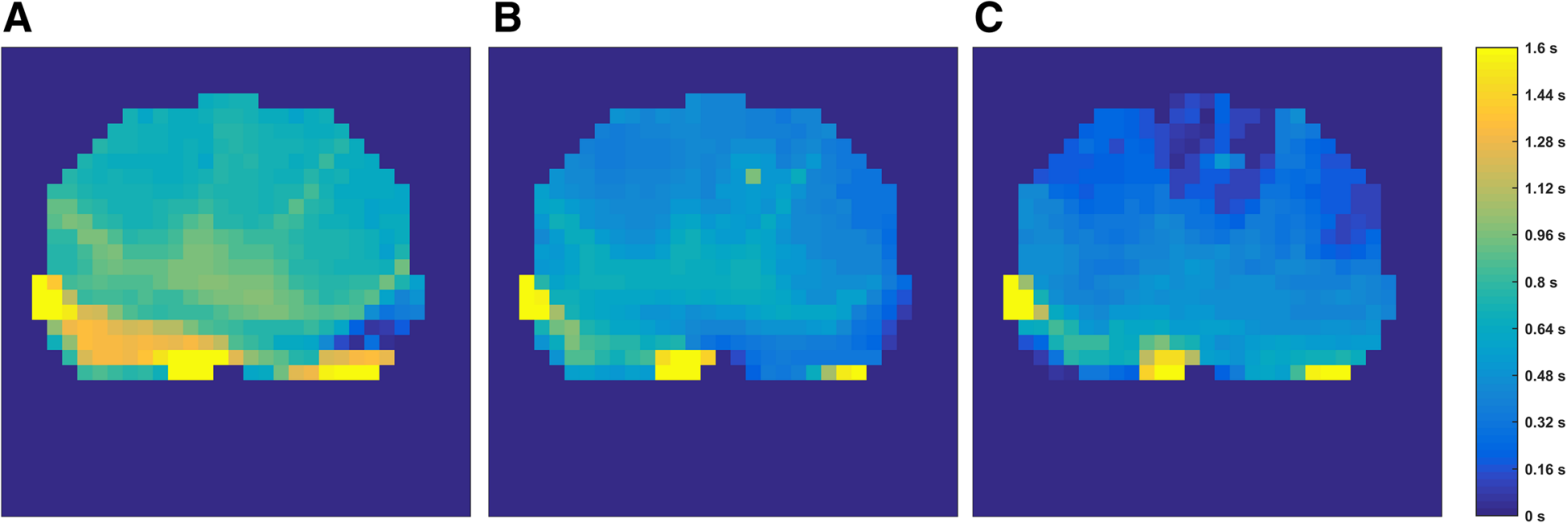
Regional ventilation delay for the injured lung under volume-controlled ventilation (VCV) (**a**), flow-controlled expiration (FLEX) ventilation (**b**) and second VCV period (**c**). Colder (blueish) colors represent lung areas that participate early in the ventilation, warmer (reddish) colors are areas with a delay in inspiration starting time. A delay in dorsal areas under VCV is visible and most likely related to the location of the lung damage. This delay is nearly compensated when FLEX ventilation is used. Thus, the originally present delayed areas participate faster under usage of FLEX ventilation, illustrating recruiting effects of FLEX in mainly dorsal areas. The beneficial effects of FLEX prevail during the second VCV period

FLEX ventilation caused a more homogeneous start of inspiration in larger lung areas in the healthy lungs compared to VCV (Fig. 4). This effect was nearly mitigated in the second VCV period. In the injured lungs, the RVD showed a delay of ventilation during VCV in the dorsal parts of the lung (Fig. 5). With FLEX, this delay in the dorsal area was reduced. In contrast to the findings in the healthy lungs, the reduced delay achieved during FLEX ventilation sustained over the second VCV period in the injured lungs.

## Discussion

The main findings of our study are that ventilation with FLEX resulted in an elevated mean airway pressure and a lower expiratory peak flow compared to VCV with otherwise identical respiratory parameters. In terms of regional ventilation, FLEX was associated with an elevated baseline in the global and in the regional impedance curves, a shortened end-expiratory zero-flow period, a redistribution of tidal ventilation from ventral to dorsal regions and a shorter and more homogeneously distributed delay in regional ventilation. The effects of FLEX were present in the healthy lungs but even more pronounced in injured lungs. These findings support our hypothesis that FLEX is associated with lung recruiting effects.

General recruitment was most intuitively reflected in the elevated impedance curve baselines when FLEX was switched on and their immediate drops when FLEX was switched off. This occurred regardless of the health condition of the lung and was evident in all regions of interest. Similar behavior of impedance curves is seen when PEEP is increased and decreased [16, 19–21]. The observed baseline shifts can therefore be attributed to an increased functional residual capacity generated by the decelerated expiration during ventilation with FLEX.

In this context, an important observation was on the lung behavior during the end-expiratory zero-flow period. During this period, convective gas movement comes to a halt and only intra-pulmonary redistribution processes occur. These however, resulted in a further decrease in the lung impedance indicating end-expiratory derecruitment, probably based on pendelluft effects and redistribution of volume out of the observation plane of the EIT measurement. With conventional VCV such derecruitment during the end-expiratory zero-flow period could only be reduced by increasing PEEP or by reducing expiration time. However, increasing PEEP would undesirably increase peak pressure and therefore increase the risk of overdistension in the non-dependent lung areas. Adjusting expiratory time might increase the risk of auto-PEEP due to incomplete expiration. By contrast, with FLEX, the end-expiratory zero-flow period per se is shorter and accordingly, the end-expiratory derecruitment was found to be less pronounced during ventilation with FLEX compared to conventional VCV. It has to be noted that the risk of generating auto-PEEP during ventilation with FLEX was thereby minimized since FLEX was set in a fashion that at the end of expiration, flow subsided completely. A second effect might be explained with respect to temporal effects of alveolar collapse, as proposed by a mathematical alveolar model [22], which postulates a population of alveoli that close “in a matter of seconds or less” after alveolar pressure is below closing pressure. The FLEX-induced slow pressure decrease results in longer maintenance of alveolar pressure above closing pressure during expiration. As a consequence the percentage of breath cycle time at which the alveolar pressure is below closing pressure is significantly lower compared to conventional passive expiration and consequently there is less time for the alveoli to collapse. In total, with FLEX the dynamics of expiration were fundamentally changed compared to passive expiration: the lungs stayed in tidal motion for a longer period of expiratory time, which we consider to be more physiological because it is closer to the spontaneous breathing pattern.

The behavior of the impedance baseline after switching from FLEX to conventional VCV is of special interest. In the healthy lungs, the global baseline levels during conventional VCV sequences before and after ventilation with FLEX were comparable. However, the baselines of the dorsal impedance curves were significantly lower in the second VCV sequence. This effect is not fully understood yet - one could speculate that due to recruitment a three-dimensional redistribution of ventilation occurred, which could not be visualized owing to the limitation of EIT to a single observation plane. Further studies would be needed to investigate this in greater depth. Contrary to this, both ventral and dorsal baselines were significantly elevated in the injured lungs during the period of conventional VCV following FLEX ventilation (VCV2) compared to the conventional VCV period before (VCV1). This shift provides evidence that the recruitment prevailed at least in the short term after FLEX was switched off and some recruitment could be sustained in the injured lung. However, since only 1 min of the second volume-controlled sequence was recorded, no prediction on long-term effects after FLEX ventilation can be given here and further studies would be required to investigate those.

If we assume that FRC is higher during FLEX ventilation than during conventional VCV, it is of special interest how this surplus is distributed within the lung. The fractional impedance distribution showed a redistribution of the ventilation to dorsal areas. Even though the majority of ventilated lung area remained in the ventral region, a significant shift towards increased ventilation in dependent parts of the lung occurred with FLEX. Ventral areas were less aerated during FLEX ventilation than during VCV ventilation whereas dorsal areas showed an increase in ventilation. This effect again occurred in the healthy and in the injured lungs and again it was more pronounced when the lung was injured. Probably, the healthy lungs had a lower potential for recruiting effects compared to the injured ones and hence the shift to dependent areas was smaller for this case.

Homogenizing effects of FLEX were impressively illustrated by the RVD. With FLEX, larger areas participated earlier in ventilation than during conventional VCV. In the injured lungs the effect was more pronounced. The delay in inspiration during conventional VCV is likely related to the location of the injured lung areas. The RVD maps drafted by Muders and colleagues displayed increased delay times in the dorsal lung regions due to end-tidal lung collapse when ventilating lung-injured pigs [18]. With FLEX ventilation, the delay is reduced in the dorsal areas, indicating faster and more homogeneous dispersal of gas in the initial inspiration phase and consequently a better recruitment state, compared to VCV.

## Limitations of the study

In this study, we recorded EIT sequences over a rather short period of time. Thus, long-term effects of FLEX ventilation cannot be deduced from this setting. However, the effects of FLEX ventilation on lung recruitment set in immediately and were well observable within our time window. We did not measure blood gases, since we did not expect noticeable changes in these within our short observation window. Nevertheless, the effects of FLEX ventilation on pH and partial pressure of arterial carbon dioxide (paCO_2_) would be important in the view of long-term ventilation. A prior study suggests that paO_2_ improves and paCO_2_ is lower during 6 h of FLEX ventilation compared to VCV [5]. In addition, our study was restricted to using only one tidal volume, one frequency and the supine position. It would be of interest to investigate the recruiting effects of FLEX with respect to systematic variation of these parameters.

Our protocol did not contain an auto-PEEP measurement. The gold standard of auto-PEEP measurement is to apply an end-expiratory pause of appropriate length (5–10 s). Such a hold maneuver would provide prolonged static conditions. As we assume that the effects of FLEX rely on a continued dynamic situation, this would immediately diminish the effects of FLEX. Therefore, such measurement was not part of our protocol. Consequently, we cannot fully exclude the generation of a minor auto-PEEP. The calculated P_0_ values were always lower than the respective PEEP. The underestimation of PEEP (in the absence of auto-PEEP) is in accordance with the findings of Eberhard and colleagues [11], who attributed this to inhomogeneity and tissue stress. Both are more effective during fast expiration since the lung tissue needs more time to reach equilibrium, than during slow expiration. Correspondingly, the values for P_0_ converged with PEEP for the longer active expiration time during FLEX ventilation; they were however always below PEEP and did thus not provide evidence for a relevant auto-PEEP. Further, the mean dynamic compliance of the healthy animals was 51 ml/cmH_2_O and of animals with lung injury 16 ml/cmH_2_O. If auto-PEEP was the reason for the observed FLEX-related volume increases of 57 ml and 115 ml, an auto-PEEP of about 2 cmH_2_O or 7 cmH_2_O would be required, respectively. We would expect that an auto-PEEP of such an amount would have caused a measurable end-expiratory flow.

The technique of slowing down the expiratory phase is not entirely new. Earlier approaches have also aimed at decelerating expiration by adding expiratory resistance [23, 24]. Although the ideas behind these approaches are similar to that of FLEX, the constant additional expiratory resistances increased expiratory time by increasing the time constant. Incomplete expiration might therefore have prevented clinically relevant benefits [24, 25]. By contrast, FLEX decouples the expiration from the time constant. It causes flow deceleration only in the early expiration phase and an improved flow rate in the late expiration phase. As a consequence, the lung volume empties in a linearized fashion and an expiratory gas flow is achieved nearly throughout expiration. Thus expiration time is not necessarily prolonged.

## Conclusion

FLEX ventilation elevated the baseline of impedance curves, shortened the no-flow phase at the end of the expiration, redistributed ventilation from ventral towards dorsal areas and homogenized the regional ventilation. The effects of FLEX were more pronounced in injured lungs. These findings support our hypothesis that FLEX ventilation has recruiting effects and indicate potential lung-protective effects of FLEX during mechanical ventilation.

## Abbreviations

ARDS: Acute respiratory distress syndrome
EIT: Electrical impedance tomography
FiO_2_: Fraction of inspired oxygen
FLEX: Flow-controlled expiration
FRC: Functional residual capacity
i.v.: Intravenous
P_0_: Dynamic auto-positive end-expiratory pressure
PaCO_2_: Partial pressure of carbon dioxide in arterial blood
PaO_2_: Partial pressure of oxygen in arterial blood
Paw: Airway pressure
PEEP: Positive end-expiratory pressure
PV: Pressure/volume
RVD: Regional ventilation delay
TV_v_/TV_d_: Tidal variation in the ventral/dorsal region
VCV: Volume-controlled ventilation
δZ: Loss of impedance during zero-flow period
